# Single-cell gene set scoring with nearest neighbor graph smoothed data (gssnng)

**DOI:** 10.1093/bioadv/vbad150

**Published:** 2023-10-18

**Authors:** David L Gibbs, Michael K Strasser, Sui Huang

**Affiliations:** Shmulevich Lab, Institute for Systems Biology, Seattle, WA 98106, United States; Huang Lab, Institute for Systems Biology, Seattle, WA 98106, United States; Huang Lab, Institute for Systems Biology, Seattle, WA 98106, United States

## Abstract

**Summary:**

Gene set scoring (or enrichment) is a common dimension reduction task in bioinformatics that can be focused on the differences between groups or at the single sample level. Gene sets can represent biological functions, molecular pathways, cell identities, and more. Gene set scores are context dependent values that are useful for interpreting biological changes following experiments or perturbations. Single sample scoring produces a set of scores, one for each member of a group, which can be analyzed with statistical models that can include additional clinically important factors such as gender or age. However, the sparsity and technical noise of single-cell expression measures create difficulties for these methods, which were originally designed for bulk expression profiling (microarrays, RNAseq). This can be greatly remedied by first applying a smoothing transformation that shares gene measure information within transcriptomic neighborhoods. In this work, we use the nearest neighbor graph of cells for matrix smoothing to produce high quality gene set scores on a per-cell, per-group, level which is useful for visualization and statistical analysis.

**Availability and implementation:**

The gssnng software is available using the python package index (PyPI) and works with Scanpy AnnData objects. It can be installed using “pip install gssnng.” More information and demo notebooks: see https://github.com/IlyaLab/gssnng.

## 1 Introduction

In biological systems, groups of genes carry out biological functions via pathways, protein complexes, and signaling cascades. It is often informative to assess the activity of these transcriptional programs through examining the concerted expression of several genes together. Their individual expression may be weak, but co-expression of genes in a pathway is often a strong indication of activity. Using gene set enrichment techniques can shed light on how pathways and modules take part in the response to perturbations (Subramanian *et al.* 2005, Hung *et al.*, 2012, Maciejewski, 2014, Maleki *et al.*, 2020).

Previously, single sample methods were developed to compute a gene set score independently for each sample. The matrix of scores (samples by gene set) are used as the basis of further analysis, such as in visualizations and statistical modeling (Barbie *et al.* 2009, Foroutan *et al.* 2018). With single sample scores, statistical models can be better specified since they can include not only gene set scores but also clinical, biospecimen, and technical variables. From single sample analysis, it is a natural extension of these methods to single-cell data.

Much of the previously mentioned methods were designed for bulk expression data, like gene microarrays and RNA-seq, where millions of cells are batch processed. These experiments provide ample measures on practically all genes, but represent an unknown mixture of cells (i.e. a population average). Single-cell transcriptomics provides more precise information on the mixture of cell types, the heterogeneity of those cells, and allows for the discovery of new subtypes. However, the data are shallow (few counts), sparse (many zeros in the expression matrix), and noisy with a much smaller collection of total genes measured in each cell (Kim *et al.* 2020). This causes difficulties in standard types of analysis, such as differential expression, but also gene set analysis. For example, methods that rely on ranked expression profiles, like ssGSEA, will be operating on data that have shared rank across the high proportion of zeros or integer collisions (e.g. genes with 1 count). Due to frequent ties, ranking becomes highly unstable; small changes in a gene’s counts lead to large changes in rank, further leading to large changes in gene set scores.

In the related task of determining differential expression, recent studies have shown that these problems can be avoided by using “pseudobulk” profiles, created by summing across groups of cells (Squair *et al.* 2021). The summation is a dimensional reduction (in the cell dimension) that creates higher abundances, lower noise floors, breaks many of the ties in expression counts, and allows for better overlap with gene sets. However, in applying this “pseudobulk” transformation, most of what makes single-cell data valued is lost; namely the heterogeneity and variability observed across cells and conditions.

Various approaches have been developed that address the noise and sparsity of single-cell data. In many instances these focus on the use of K-nearest neighbor graphs (KNNs), which are a fundamental part of single-cell data analysis, making them an attractive target in algorithm development (Lun *et al.* 2016, Wagner *et al.* 2017). MAGIC (Dijk *et al.* 2018) involves modeling the lower dimensional data manifold through diffusion on the KNN graph, while MetaCell (Baran *et al.* 2019) makes partitions on subgraphs to model an archetypal cell. A methods variation is found in scGSEA where document modeling followed by non-negative matrix factorization are used instead of KNNs (Franchini *et al.* 2023).

With these approaches in mind, we have developed a python package that works with Scanpy AnnData objects to produce a gene set score for each cell (Wolf *et al.* 2018). Included are a collection of scoring functions from previously described single sample methods, similar to decoupleR (Badia-I-Mompel *et al.* 2022), and functions that ingest gene sets from standard .gmt files or from OmniPath (Türei *et al.* 2016).

## 2 Overview of the method

**Figure 1.**
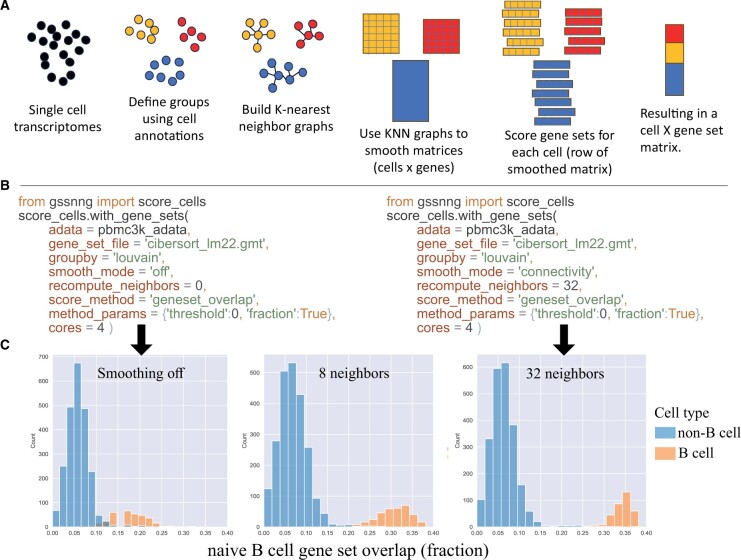
(A) Overview of the approach. (B) Code examples to generate the results shown in next panel. (C) Effect of neighborhood smoothing for PBMCs and a B-cell signature. Gene set overlap (cells from 10× 3kPBMC dataset) with smoothing turned off, or K-nearest neighbor smoothing (*K* = 8 or *K* = 32). B cells in the dataset (smaller distribution on the right) start with ∼15% of their measured genes overlapping with the “B.cell.naive signature.” After data smoothing the gene set overlap grows to over 30%.

Briefly, cells are grouped using user-specified attributes such as cell type, cluster label, batch, or condition. These groups are used to define collections of cells that form disjoint nearest neighbor graphs. After creating groups, the remainder of the process is done in parallel by group. The graphs, represented as a matrix, are used in smoothing the gene expression counts matrix. Lastly, the smoothed expression profile for each cell is passed to a selected gene set scoring function (Fig. 1A).

## 3 Grouping cells with the “groupby”

When analyzing a combined or integrated dataset (e.g. contains several samples, patients, or batches), it may be beneficial to first group cells into phenotypically similar subsets before building the KNN graphs. The “groupby” parameter, is a list that maps to a set of categorical variables in the AnnData.obs table, and is used to sort the cells into chunks that can be processed in parallel. We use python’s multiprocessing starmap function to asynchronously process each “groupby-group.” This leads to a set of smoothed count matrices that are each specific to a selected phenotype. For example, one might group cells by cluster label, so smoothing is constrained to transcriptionally similar cells.

## 4 The nearest neighbor graph

After grouping, a nearest neighbor graph of cells is constructed using scanpy.pp.neighbors, which in turn uses PyNNDescent (Dong *et al.* 2011). To calculate distances between cells and to determine nearest neighbors, we use a density-adjusted Gaussian kernel, commonly used in graph based clustering and dimension reduction of scRNAseq (McInnes *et al.* 2018). The choice of K in building the KNN, will be context dependent, but in practice, setting K to around 32 works well. One suggestion to determine K, is to use the “geneset_overlap” function, which returns the size of the intersection between the gene set and the expressed genes (above a given threshold). When the “geneset_overlap” score plateaus, the limitations of the data become apparent (see Fig. 1).

## 5 Matrix smoothing

To address the noisy and sparse nature of single-cell expression data, we apply nearest neighbor smoothing to produce a smoothed gene expression profile for each cell based on its neighbors. This assumes that gene expression varies smoothly along the data manifold (approximated by the nearest neighbor graph) and hence we can use information from neighboring cells to denoise the expression profiles of cells (similar to Gaussian smoothing in images, which assumes that pixel intensities vary smoothly in space) (Shapiro and Stockman 2001, Ronen and Akalin 2018). The smoothed expression matrix of cells by genes is calculated via matrix multiplication AX=M where *A* represents a binary adjacency matrix or a weighted matrix of connectivities, and *X* is the cell by gene matrix of gene expression counts. Additionally, one can set the “smooth_mode” to “off” in order to disable smoothing. As both A and X are typically sparse, our implementation uses the scipy sparse matrix library as an effort to be mindful of memory use (Becht *et al.* 2018).

## 6 Scoring functions

As part of the package, several functions are available for gene set scoring. These include “singscore” (Foroutan *et al.* 2018), “ssgsea” (Barbie *et al.* 2009, Abazeed *et al.* 2013), “rank_biased_overlap” (Webber *et al.* 2010), “mean_z_score,” “average_score,” “median_score,” “summed_up” (Pont *et al.* 2019), and a “geneset_overlap” count. Descriptions of each function are found in the Supplementary Material, Section 3. For each gene set, the gene set scores are recorded in the AnnData.obs pandas table, with one column per gene set, facilitating visualization through compatibility with the Scanpy plotting system.

## 7 Validation of GSSNNG

In order to validate the method, we used three datasets with known “ground truths.” First, we compare smoothed and non-smoothed gene set scoring to identify B cells in a mixture of peripheral blood mononuclear cells (PBMCs) (Genomics 2016). Second, we show how smoothed gene set scoring helps to identify cells with an immune response in a dataset of phagocytes treated with lipopolysaccharide (LPS) (Squair *et al.* 2021). Third, we assess smoothed and non-smoothed gene set scoring of cellular response genes in a dataset of endothelial cells exposed to spinal injury in mice (Hagai *et al.* 2018).

### 7.1 10× Genomics pbmc3k

In this dataset, PBMCs from a healthy donor were previously sequenced and annotated with cell type labels. The pre-processed data contains 2,638 cells with 1,838 genes. We applied the LM22 gene sets (cell type signatures) to produce cell type specific scores (Chen *et al.* 2018), focusing on the naive B cell signature (Fig. 1B). The “geneset_overlap” function was applied to non-smoothed data and smoothed data (*K* = 8 or *K* = 32 neighbors). This function returns the number (or fraction) of genes that have expression measures above the given threshold. The expectation is that the B-cell score distribution would show separation between cells labeled as B cells and other types of cells. The results show that for previously annotated B cells the number of genes from the B.cell.naive signature was effectively doubled with smoothing. With the limited set of genes available, the increase in gene set overlap does not improve noticeably past 32 neighbors. But clearly, through the use of matrix smoothing, the B-cell specific signal was improved without an effect to non-B cells and allows one to easily distinguish B cells from other cells using the signature scores.

### 7.2 Hagai *et al.*

**Figure 2.**
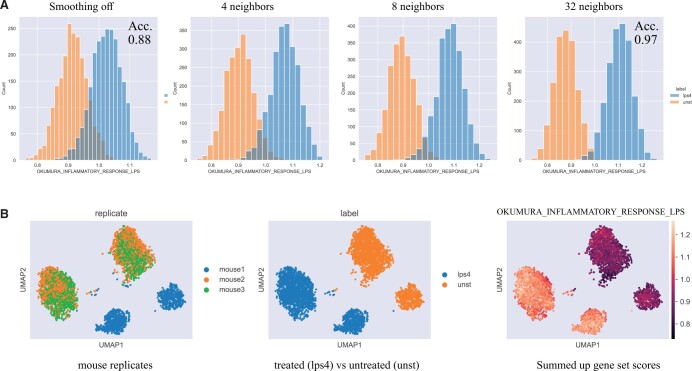
(A) Murine phagocytic cells were scored using the “Okumura inflammatory response LPS” gene set and the “summed_up” function. The histogram shows that by increasing the size of the neighborhood, the gene set score distributions separate between treated (right) and untreated (left) groups. Using the median score as a cut point on labels (treated vs untreated), the accuracy was improved from 0.88 to 0.97 with smoothing. (B) UMAP plot of the cells in (A).

Murine phagocytic cells were treated with LPS which causes a strong immune response. The ”Okumura Inflammatory Response LPS” gene set (Okumura *et al.* 2003) contains genes related to this cellular warning system (see Fig. 2). It is expected that cells treated with LPS will show an immune response, and that with smoothing, the distribution of immune response scores would differentiate treated from untreated cells. The dataset was subsampled to 2,340 lps4 treated cells and 2,104 untreated cells and scored using the “summed_up” ranks function, which simply sums up the smoothed and ranked expression of signature genes for each cell. The results are compared between no smoothing and smoothing with 4, 8, or 32 neighbors. It was observed that with increasing neighborhood size, the separation in the distribution of gene set scores increased, improving the prediction of treatment group (Fig. 2). To more precisely quantify the ability of the score to distinguish the treatment groups, we calculated the area under the curve (AUC) using sklearn’s roc_auc_score function, the AUC was calculated to be 0.95 for unsmoothed data, and improved to 1.0 for smoothed data. When making a prediction of LPS treatment using the median score as a cut point on labels, the accuracy was 0.88 for nonsmoothed data and 0.97 for smoothed data.

### 7.3 Squair *et al.*

**Figure 3.**
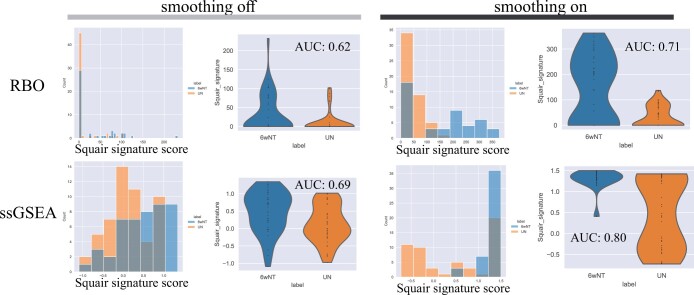
Endothelial cells from control mice (UN, right) and mice who received spinal injuries (6wNT, left) show a response to hypoxic environment. Scores are shown from no smoothing and smoothing with 32 neighbors using two methods, rank biased overlap (RBO) and single sample GSEA (ssGSEA). The AUC in predicting the cell label was improved with both scoring functions (0.62 to 0.71 for RBO and 0.69 to 0.80 for ssGSEA).

In this experiment, mice received spinal injuries provoking a cellular response. The study reported that from all cells investigated, endothelial cells showed the greatest response. From the differential expression analysis on endothelial cells, 19 genes were validated using RNAscope. Because not all genes were validated, the gene sets were constructed from 12 of the 19 genes, with 7 showing higher expression in the injured mice and 5 showing lower expression for injured mice, thus making a two part gene set (both up and down). Gene set scoring was performed using the “rank biased overlap (RBO)” and “ssGSEA” functions on ranked expression on endothelial cells, and it was observed that with smoothing the gene set score distribution for injury response shifted towards higher values, more clearly defining the injured and the control groups (Fig. 3). In predicting exposure, the area under the curve (AUC) was 0.62 and 0.69 using unsmoothed data, which improved to 0.71 and 0.8 with smoothed data after applying the RBO and ssGSEA score functions, respectively.

### 7.4 Smoothed scores are robust to downsampling

Our initial hypothesis was that the inherent measurement noise in scRNAseq makes gene-set scoring challenging and smoothing gene expression along the nearest neighbor graph counteracts this to some extent. Here, we support this claim by introducing additional measurement noise to existing data via downsampling the count matrices. This leads to additional variance in the counts and increases the amount of zeros in the count matrix. In particular, we subsampled the count matrix of the PBMC dataset (from 95% to 5% of total counts), reprocessed the data using the “Zhang recipe” (Zheng *et al.* 2017) and scored each cell for the “B cells naive” signature using either smoothed or non-smoothed downsampled expression values. To quantify the effect of downsampling we calculate the AUC in predicting B cells from non-B cells (see Supplementary Fig. S1). For non-smoothed data, the AUC dropped immediately with downsampling, while for 32 neighbor smoothed data, the AUC remained over 0.95 until <10% of total counts were reached, showing the method to be highly robust.

## 8 Conclusions and limitations

There is no single and best solution to the problem of sparsity and noise in single-cell transcripomics. The method selected should depend on the question at hand and the particularities of the data. Along with data transformations like normalization, smoothing methods have the potential to remove biological variability from data. Nonetheless, gssnng provides a robust toolbox that is compatible with AnnData objects and includes nearest neighbor smoothing with a selection of gene set scoring methods.

## Supplementary Material

vbad150_Supplementary_Data

## Data Availability

The python package is available on github (https://github.com/IlyaLab/gssnng) and the PyPI index (https://pypi.org/project/gssnng/).
