# Supplementary material for: Single-cell gene set scoring with nearest neighbor graph smoothed data (gssnng)
Source: Bioinform Adv. 2023 Oct 18;3(1):vbad150. doi: 10.1093/bioadv/vbad150 (PMC10599965; doi:10.1093/bioadv/vbad150)
Supplement: vbad150_Supplementary_Data [file vbad150_supplementary_data.pdf]

GSSNNG Supplementary Materials

David L Gibbs,[1,*] Michael K Strasser,[2] and Sui Huang[2]
[1] *Shmulevich Lab, Institute for Systems Biology, 401 Terry Ave N, 98106, WA, USA*

[2] *Huang Lab, Institute for Systems Biology, 401 Terry Ave N, 98106, WA, USA*
[*] *david.gibbs@isbscience.org*

Supplementary Materials for the manuscript: "Single cell gene set scoring with nearest neighbor graph smoothed data (gssnng)." .

1. Effect of downsampling on single cell scoring

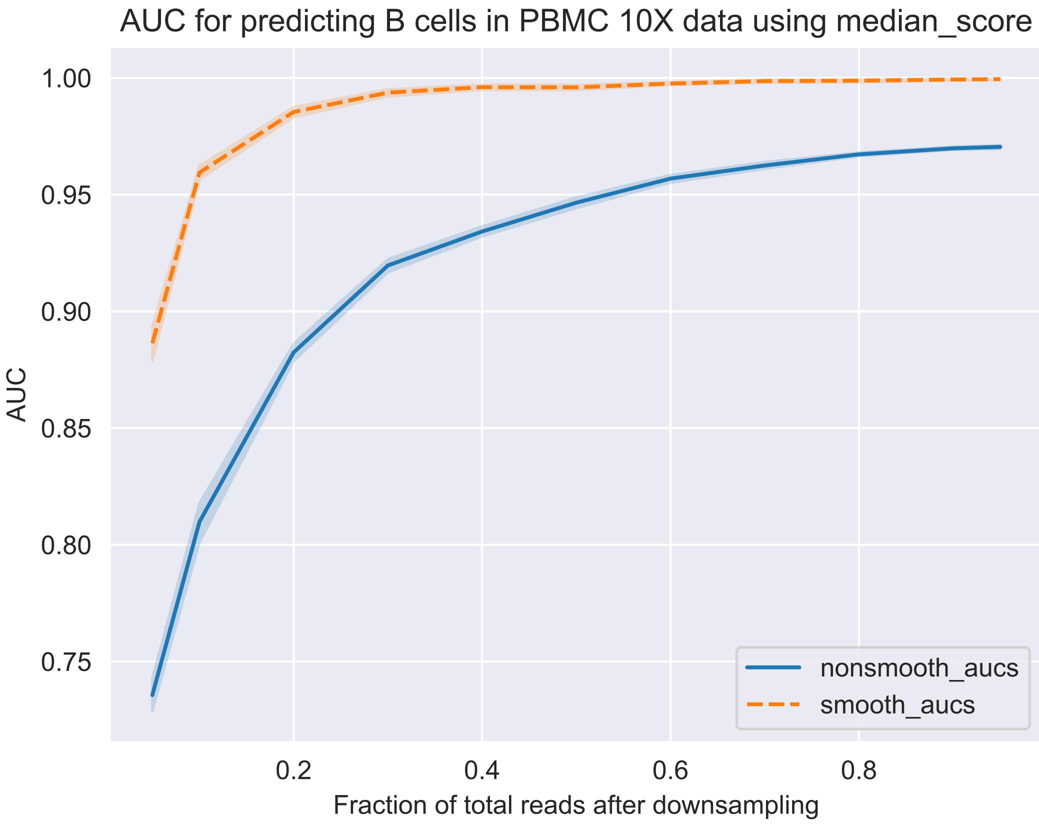

Figure S1. Predicted B cell label AUC with and without 32N smoothing. The procedure was run 20 times, with random downsampling in each iteration, error bounds are shown in the plot.

To show the effect of downsampling on gene set scores, the pbmc data was downsampled using scanpy.pp.downsample_counts so that the total number of raw counts were a percentage of the total. With and without smoothing, we downsampled the total number of raw counts in the data set at 11 points from 95% to 5% and reprocessed the data using the Scanpy Zhang et al. recipe. At each step, cells were scored using the "median_score" method, and the "B cells naive" score was used to calculate the AUC in predicting B cells from non-B cells (Figure S1). The prodcedure was run 20 times, with random downsampling each time, error bounds are shown in the plot. For non-smoothed data, the AUC dropped immediately with downsampling, while for 32 neighbor smoothed data, the AUC remained over 0.95 until less than 10% of total counts were reached, showing the method to be highly robust.

2. Gene set file formatting and naming

There are two ways to use the package, the first involves using gene set files specified with the .gmt format. Secondly, we have provided a connection to omnipath with an interface that works like decoupler.

To get signatures from omnipath, we provide the 'genesets_from_decoupler_model' function as well as a notebook (gssnng_decoupler.ipynb) that can be found in the github repository. The 'run_gssnng' function would be used, similar to the decoupler syntax.

Gene sets can also be provided using the .gmt file format, which lists one gene set per line. We generally follow the convention in single cell analysis where genes are encoded using HUGO symbols. Gene sets can be used in at least three ways according to whether one expects (or is interested in) genes being highly expressed, gene having very low expression, or extreme in general where the genes would be organized into two sets, one high and one low. In practice, the three modes of expectation can be accessed by including an '_UP' or '_DN' onto the end of the gene set name, similar to the MSigDB style of gene set naming. When it's expected that the genes within a given set will be both high and low expressed, they are split into two gene sets, processed separately, then summed. GSSNNG identifies gene sets that are paired (up + down) by matching names. They can appear in any order in the .gmt file, but the prefixes must be identical, the only difference being '_up' and '_dn' as a suffix. By default, if a gene set name doesn't have a up or down suffix, it's taken as an upregulated gene set.

To score cells with a .gmt file, please see the quick start notebook, found in the repo readme (gssnng_quick_start.ipynb), where the 'score_cells' function is used.

3. Scoring functions

For a given cell i, X_i is the vector of values which are either gene expression counts or ranked values. Then our gene set G_k is a set of indices into X_i.

Our goal is producing a score per cell, S_{ik}, where i indicates the cell and k indicates the gene set G_k.

3.0.1. Summed up

Here we sum up the ranks or counts.

$$S_{ik} = \sum_{j \in G_k} X_{ij}$$

3.0.2. Average Score

The average score takes the mean of counts or ranks given the gene set.

$$S_{ik} = \frac{1}{|G_k|} \sum_{j \in G_k} X_{ij}$$

3.0.3. Median score

Similarly, the median score takes the median of counts or ranks for the gene set.

$$S_{ik} = \text{median}(X_{ij})$$

for $j \in G_k$

3.0.4. Mean Z

The score is based on computing a Z score for each gene in the gene set, and then taking the mean over Z scores. Here it's recommended to work with unranked data.

$$S_{ik} = \frac{1}{|G_k|} \sum_{j \in G_k} (X_{ij} - \mu_i)/\sigma_i$$

where $\mu_i = \text{mean}(X_i)$ and $\sigma_i = \text{stddev}(X_i)$

3.0.5. Rank Biased Overlap

Scores derived using rank biased overlap (RBO) are generated by considering the overlap of a gene set (or other set of items) with the top n ranked genes in the list. The overlap is summed as j ranges from a lower limit (default 1) to a set parameter value, such as 50. As we descend in the ranking, a weighting of 1/l is multiplied to the size overlap, so the value produced and accumulated is futher reduced with each step down the ordered ranks. It could be described as the weighted average of agreement between sorted ranks and gene set.

$$S_{ik} = \sum_{l=1..n} l^{-1} |H_l \cap G_k|$$

where H_l is the top l number of genes after rank ordering the values in X_i.

3.0.6. ssGSEA

This method is born from GSEA (4,19), where empirical distributions of genes, both within and outside of the gene set are compared.

$$S_{ik} = \sum_{n=1..N} P_{G_k}(G_k, R_j, n) - P_{NG_k}(G_k, R_j, n)$$

where N is the total number of genes, R_i is rank ordered genes for cell i. Then the two probability distributions are defined as

$$P_{G_k}(G_k, R_j, n) = \frac{\sum_{(m \in G_k, m<n)} R_{im}^{\omega}}{\sum_{m \in G_k} R_{im}^{\omega}}$$

and

$$P_{NG_k}(G_k, R_j, n) = \frac{\sum_{(m \notin G_k, m<n)} 1}{N - |G_k|}$$

where ω is a tuning variable, by default this should be set to 0.75.

3.0.7. SingScore

SingScore (18) is a single sample gene set scoring software that was developed for use with bulk RNA-seq data. It represents an improved and extended version of ssGSEA. Generally, it can be described as a normalized mean of median centered ranks.

$$S_{ik} = \frac{\sum_{j \in G_k} R_{ij}}{|G_k|}$$

The reported SingScore is normalized by either a theoretical normalization scheme or a simplification (standard normalization) which divides the score by the total number of genes. For the theoretical normalization,

$$\bar{S}_{ik} = \frac{S_{ik} - S_{min}}{S_{max} - S_{min}}$$

where $S_{min} = (|G_k| + 1)/2$ and $S_{max} = (2N - |G_k|)/2$, that N is the total number of genes in X_i and $|G_k|$ is the size of the gene set.

4. Code Example

The following code example takes the 10X genomics pbmc3k data set and scores it for cell types using the LM22 signatures. With the scores, they can be visualized using the plotting functions from Scanpy. Please see: www.github.com/IlyaLab/gssnng for more details and notebooks with examples.

```
# First let’s install the package from github
# and clone the repo to get the example data.
!pip install git+https://github.com/IlyaLab/gssnng
!git clone https://github.com/IlyaLab/gssnng

from gssnng import score_cells
import scanpy as sc
import matplotlib
%matplotlib inline

## !! set up for google colab notebook !! ##
# these file paths point to the cloned repo from above in a google colab environment #
gene_set_file = ’/content/gssnng/gssnng/test/data/cibersort_lm22.gmt’

# read in the 10x genomics example data set
adata = sc.datasets.pbmc3k_processed()

# Then we’ll produce a gene set score for each cell,
# for each gene set in the .gmt file.
# Since groups run in parallel, set the cores parameter as appropriate.

score_cells.with_gene_sets(
        adata=adata,
        gene_set_file=gene_set_file,
        groupby="louvain",
        smooth_mode=’connectivity’,
        recompute_neighbors=32,
        score_method="singscore",
        method_params={’normalization’:’standard’},
        ranked=True,
        cores=6
    )


# Now we can visualize the scores
sc.pl.umap(adata, color=[’T.cells.CD8.up’,’B.cells.naive.up’,’louvain’],
           wspace=0.1, colorbar_loc=None)
```
